# *TGFBI* gene mutations in a Korean population with corneal dystrophy

**Published:** 2012-07-20

**Authors:** Kyong Jin Cho, Jee Won Mok, Kyung Sun Na, Chang Rae Rho, Yong Soo Byun, Ho Sik Hwang, Kyu Yeon Hwang, Choun-Ki Joo

**Affiliations:** 1 Dankook University Hospital, Department of Ophthalmology, Cheonan, Korea; 2 Seoul St. Mary’s Hospital, Department of Ophthalmology and Visual Science, College of Medicine, The Catholic University of Korea, Seoul, Korea; 3 Daejeon St. Mary’s Hospital, Department of Ophthalmology, College of Medicine, The Catholic University of Korea, Daejeon, Korea; 4 Catholic Institute for Visual Science, College of Medicine, The Catholic University of Korea, Seoul, Korea

## Abstract

**Purpose:**

To investigate the clinical and genetic features of Korean patients with corneal dystrophies associated with mutations in the human transforming growth factor-β-induced (*TGFBI*) gene.

**Methods:**

In this study, 387 subjects (71 families and 89 individuals - 268 patients having *TGFBI* corneal dystrophies and 119 normal relatives) were assessed. All subjects underwent a complete ophthalmologic evaluation, including biomicroscopic inspection and dilated fundus examination. As a control, 100 individuals without corneal disease were selected from the general population. The polymerase chain reaction (PCR) and direct sequencing were used to screen for mutations in *TGFBI*.

**Results:**

All subjects recruited exhibited a range of corneal dystrophies, including Thiel-Behnke corneal dystrophy (TBCD, R555Q; 6 families and 4 individuals), granular corneal dystrophy type 2 (GCD2, R124H; 61 families and 80 individuals), lattice corneal dystrophy (LCD; 4 families and 5 individuals; 7 with type 1 [R124C], and 2 with a variant [L527R, P542R]). The disease showed an autosomal dominant inheritance pattern in all families.

**Conclusions:**

R124H in GCD2 was the most common mutation. GCD1 and Reis-Bucklers corneal dystrophy were not found. In the GCD2 patients there were a large number of laser refractive surgery-induced corneal opacities. A spontaneous R124H mutation was confirmed in an already mutated allele that resulted in a change from a heterozygous into a homozygous form. Also, a novel mutation, P527R, was identified in LCD.

## Introduction

Corneal dystrophies, a group of inherited, bilateral, symmetric, slowly progressive corneal diseases without any relationship to environmental or systemic factors, have been mapped to 10 different chromosomes: 1, 2, 5, 9, 10, 12, 16, 17, 20, and X [1]. Affected individuals experience diminished corneal sensitivity, the sensation of the presence of a foreign body (if corneal erosions occur), halos around lights, and progressive visual impairment between the third and fifth decades of life. The majority of corneal dystrophies recognized to-date exhibit an autosomal dominant mode of transmission with a high degree of penetrance [2].

Mutations in the transforming growth factor beta-induced (*TGFBI*) gene (OMIM 601692), have been identified in several autosomal dominant (AD) corneal dystrophies, including: granular, lattice (excluding type II), Avellino, Bowman layer type I and II, and basement membrane [3,4]. *TGFBI* encodes for TGFBI-associated protein (TGFBIp), an extracellular matrix protein [5] that plays a role in cell adhesion. Corneal opaciﬁcation results largely from the deposition of aberrantly processed mutant proteins [6]. Extracellular deposition of the insoluble protein aggregates, amyloid and/or hyaline, within the cornea is the hallmark of inherited corneal disorders caused by mutations in *TGFBI*. More than 50 distinct disease-causing mutations in *TGFBI* have been identified [7]. Recent reports have demonstrated the utility of mutational analysis in defined populations (Japan, Ukraine, Mexico, Vietnam, New Zealand, Polish, Taiwan) [8-13] for identification of unique phenotypic variants, novel mutations and genotype-phenotype correlations. An apparent genotype–phenotype correlation has emerged from these molecular studies; four distinct heterozygous recurrent mutations were found to be associated with four specific phenotypes: p.R555W in granular corneal dystrophy type 1 (GCD1), p.R124C in lattice corneal dystrophy type 1 (LCD1), p.R124H in granular corneal dystrophy type 2 (GCD2), and p.R555Q in Thiel-Behnke corneal dystrophy (TBCD).

In this paper, we present the results of a clinical and genetic analysis of a Korean population of patients with *TGFBI*-related corneal dystrophies. Novel *TGFBI* mutations and previously unrecognized genotype–phenotype correlations were identified in this population.

## Methods

This study was performed in accordance with the Declaration of Helsinki, and written informed consent was signed by all subjects or their parents. The study design was approved by the Institutional Review Board of the Seoul St. Mary’s Hospital, Catholic University, Seoul, Korea.

### Patients

Initially, 387 subjects (71 families and 89 individuals - 268 patients having *TGFBI* corneal dystrophies and 119 normal relatives) were assessed. The mean age of the patients was 41 years (±20 years). Seventeen patients had undergone a refractive surgery procedure in the past.

### Phenotype analysis

A detailed slit-lamp biomicro-examination was performed and photographs were obtained. In addition, detailed genealogical information, including surname and birthplace analysis, was collected to exclude relationships among the families. Available consenting first and second degree relatives were included, their clinical status determined and pedigree charts constructed. We analyzed corneal change patterns, location, symmetry and progression with age. In addition, the heterogeneity of corneal morphology among the patients with identical *TGFBI* mutations was assessed.

### Histologic examination

The whole corneal button from a patient with a novel *TGFBI* mutation after penetrating keratoplasty was processed by standard methods involving sectioning of the tissue samples. Corneal sections were stained with hematoxylin and eosin (HE), periodic acid-Schiff (PAS), and Congo red and further analyzed by light microscopy for the presence of amyloid deposits.

### DNA collection, isolation, amplification and sequencing

Genomic DNA was extracted from blood samples using a genomic DNA miniprep kit for blood (Axygen, Union City, CA). The DNA fragments that encode portions of the *TGFBI* gene were amplified by polymerase chain reaction (PCR). The primers for PCR are shown in Table 1. PCR was performed with 25 ng of genomic DNA as a template in a mixture of PCR buffer, 2.5 mM MgCl_2_, 200 nM dNTPs, 0.4 pM of each primer, and 0.75 U of h-Taq polymerase (Solgent, Daejeon, Korea; Table 1) [14]. For DNA sequencing, amplified DNA was purified (QIAquick PCR purification kit; Qiagen, Les Ulis, France) and sequenced on a 3730 xl automated DNA sequencer (Applied Biosystems, Foster City, CA). Nucleotide sequences were compared with the wild-type *TGFBI* sequence (GenBank NG_012646.1).

**Table 1:** Primers for *TGFBI* amplification.

**Exons**	**Primers**	**(5′→3′)**	**Amplicons (bp)**
1	BIGH3–1F	GCTTGCCCGTCGGTCGCTA	234
	BIGH3–1R	TCCGAGCCCCGACTACCTGA	
2	BIGH3–2F	AGGCAAACACGATGGGAGTCA	204
	BIGH3–2R	TAGCACGCAGGTCCCAGACA	
3	BIGH3–3F	CCAGATGACCTGTGAGGAACAGTGA	232
	BIGH3–3R	CCTTTTATGTGGGTACTCCTCTCT	
4	BIGH3–4F	TCCCTCCTTCTGTCTTCTGC	273
	BIGH3–4R	AGACTCCCATTCATCATGCC	
5	BIGH3–5F	CCTGGGCTCACGAGGGCTGAGAACAT	387
	BIGH3–5R	GCCCCTCTTGGGAGGCAATGTGTCCC	
6	BIGH3–6F	CCTGGGCTCACGAGGGCTGAGAACAT	403
	BIGH3–6R	GCCCCTCTTGGGAGGCAATGTGTCCC	
7	BIGH3–7F	GTGAGCTTGGGTTTGGCTTC	387
	BIGH3–7R	ACCTCATGGCAGGTGGTATG	
8	BIGH3–8F	TGAGGTTATCGTGGAGTG	435
	BIGH3–8R	CACATCAGTCTGGTCACA	
9	BIGH3–9F	ACTCACGAGATGACATTCCT	284
	BIGH3–9R	TCCAGGGACAATCTAACAGG	
10	BIGH3–10F	TAGAAGATACCAGATGTTAAGG	426
	BIGH3–10R	TGTCAGCAACCAGTTCTCAT	
11	BIGH3–11F	GGATAATGACCCTGCTACATGC	330
	BIGH3–11R	TCCCCAAGGTAGAAGAAAGC	
12	BIGH3–12F	CATTCCAGTGGCCTGGACTCTACTATC	318
	BIGH3–12R	GGGGCCCTGAGGGATCACTACTT	
13	BIGH3–13F	CCTCCTTGACCAGGCTAATTAC	299
	BIGH3–13R	GGCTGCAACTTGAAGGTTGTG	
14	BIGH3–14F	CTGTTCAGTAAACACTTGCT	261
	BIGH3–14R	CTCTCCACCAACTGCCACAT	
15	BIGH3–15F	CCCTCAGTCACGGTTGTT	348
	BIGH3–15R	GGAGTTGCCTTGGTTCTT	
16	BIGH3–16F	CTTGCACAACTTATGTCTGC	319
	BIGH3–16R	TGCACCATGATGTTCTTATC	
17	BIGH3–17F	AGTGAAGTTTCACAAACCAC	475
	BIGH3–17R	CCACATTTGGGATAGGTC	

## Results

All subjects recruited (Table 2) had a range of corneal dystrophies including Thiel-Behnke corneal dystrophy (TBCD), granular corneal dystrophy type 2 (GCD2), Lattice corneal dystrophy (LCD; type 1 and variants). The disease showed an autosomal dominant inheritance pattern in all families.

**Table 2:** Corneal dystrophy phenotypes and mutation analysis in *TGFBI* (5q31).

**Corneal dystrophy**	**Exon**	**Nucleotide change**	**AA change**	**Number of families**	**Number of individuals**	**De novo**
TBCD	12	c.1664G>A	R555Q	6	4	
GCD2	4	c.371G>A	R124H	61	80	
LCD1	4	c.370C>T	R124C	2	5	
Variant LCD	12	c.1580T>G	L527R	1		
	12	c.1625C>G	P542R	1		Yes
Total				71	89	

TBCD: Thiel-Behnke corneal dystrophy; GCD2: Granular corneal dystrophy, type 2; LCD1: Lattice corneal dystrophy, type 1; Variant LCD: Variant lattice corneal dystrophy.

### 

#### Thiel-Behnke corneal dystrophy

**Figure 1:**
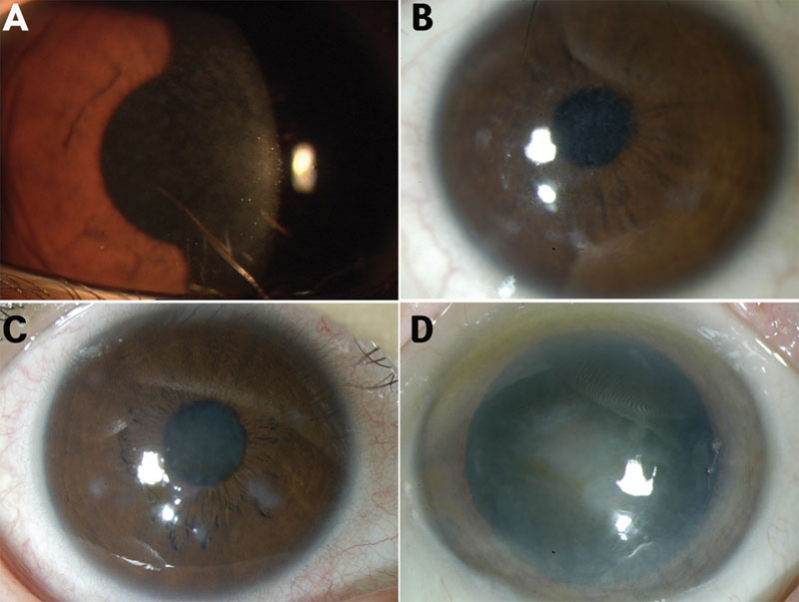
Phenotypes of Thiel-Behnke corneal dystrophy (all carried the R555Q mutation of the heterozygous form). A: An 11-month-old female, irregularly shaped scattered opacities with non-involvement of the peripheral cornea in the Bowman layer. **B**: A 26-year-old female, subepithelial reticular (honeycomb) opacities with non-involvement of the peripheral cornea. **C**: A 43-year-old female, opacities united, resulting in denser and larger opacities. **D**: A 69-year-old female, opacities, progressing to the deep stromal layers and corneal periphery.

Six families and four individuals were identified with ‘typical’ TBCD. All patients carried the R555Q mutation (heterozygous form). The mean age of this group of patients was 31 years (±20 years, range 1–78). The symptoms or signs were initially observed during childhood. An 11-month-old female patient who was otherwise in good medical health presented with corneal abrasion. She had not experienced any corneal trauma. Bilateral, irregularly shaped scattered opacities with peripheral cornea non-involvement in the Bowman layer were observed by slit-lamp examination (Figure 1A).

Recurrent corneal erosions caused ocular discomfort and pain in the first and second decades. Gradual visual impairment developed later. Two patients were suffering photorefractive keratectomy due to decreased vision in the sixth decade. When these patients were younger, symmetric subepithelial reticular (honeycomb) opacities did not involve the peripheral cornea. These opacities united, forming denser and larger opacities, and progressed to deep stromal layers and the corneal periphery with advancing years. Vision deteriorated progressively due to increasing corneal opacification (Figure 1B-D).

#### Granular corneal dystrophy, type 2

**Figure 2:**
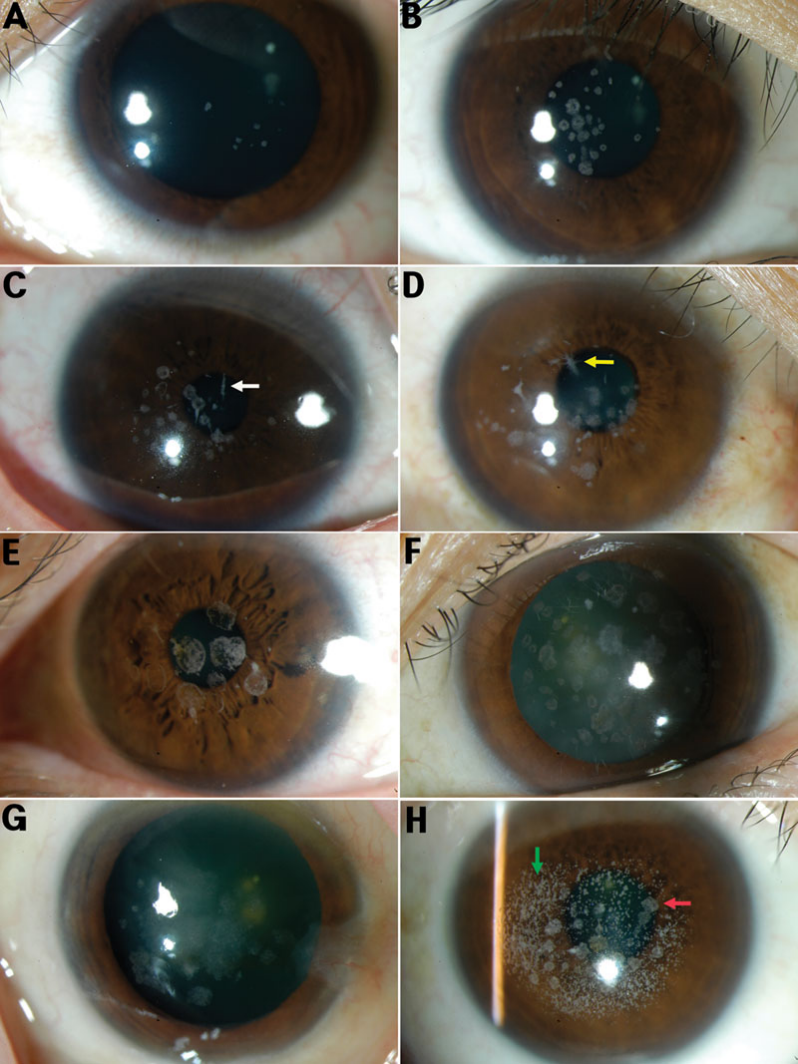
Phenotypes of granular corneal dystrophy, type 2 (all carried the R124H mutation). **A**: A 16-year-old male, subtle superficial stromal tiny whitish dots. **B**: A 24-year-old female, rings or stellate-shaped snowflake stromal opacities appeared between the superficial stroma and the mid stroma. **C**: A 34-year-old male, lattice lines in the deeper cornea (white arrow). **D**: A 47-year-old female, star-shaped stromal opacities (yellow arrow). **E**: A 55-year-old male. **F**: A 65-year-old female, more superficial, translucent, flattened breadcrumb opacities. **G**: A 77-year-old female, opacities coalesced in the anterior stroma. **H**: A 39-year-old female with a history of LASIK surgery, diffuse, confluent, white, and small opacities (green arrow) coexisted with discrete and granular opacities (red arrow).

Sixty-one families and 80 individuals were identified with GCD2. All patients carried the R124H mutation (including the homozygous form in 11 patients). The mean age of this group of patients was 44 years (±20 years, range 6–83). The onset of symptoms (most commonly decreased vision) was most often observed in the fifth or sixth decades and initial signs (incidentally discovered corneal opacities) were observed in the third or fourth decades. Homozygous patients were diagnosed as early as six years of age. Seven patients had received keratoplasty due to decreased vision (mean age 45 years). Eighteen patients had a history of laser refractive surgery (photorefractive keratectomy [PRK] 6, laser-assisted in situ keratomileusis [LASIK] 12). During their teenage years, slit-lamp examinations showed signs of subtle tiny whitish dots on the superficial stroma. During the next stage of the condition (third decade), rings or stellate-shaped snowflake stromal opacities became evident between the superficial stroma and the mid stroma. In the fourth and fifth decades, star-shaped stromal opacities appeared among disk-shaped opacities and some patients demonstrated lattice lines in the deeper cornea. In the final stage, there was a more superficial, translucent, flattened breadcrumb opacity, which coalesced in the anterior stroma. Granular opacities were formed by gradual aggregation of surrounding fine deposits based on clinical observation. The patients’ vision progressively deteriorated due to the increasing corneal opacification (Figure 2A-G).

In the patients with a history of laser refractive surgery, the morphological features of corneal deposits were different. Deposits after laser ablation appeared mainly within the ablation zone and showed diffuse, confluent, white, and small opacities. In most cases, these opacities coexisted with discrete and granular opacities that may not have been associated with refractive surgery (Figure 2H).

**Figure 3:**
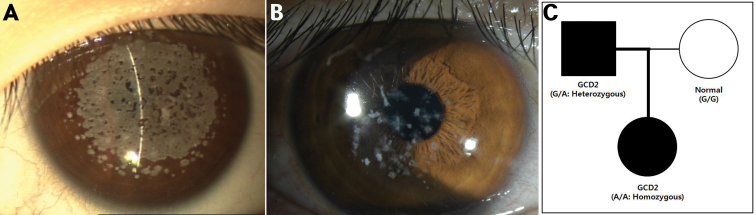
Family with granular corneal dystrophy, Type 2, in which a spontaneous R124H mutation was added to an already mutated allele, resulting in a change from a heterozygous to a homozygous form. **A**: A 6-year-old female (proband, homozygous form), dense and confluent opacities with peripheral cornea non-involvement in the stromal layer. **B**: A 32-year-old male (father of proband, heterozygous form), star- and disc- shaped opacities. **C**: Pedigree, homozygous R124H mutation (CGC→CAC), heterozygous R124H mutation, and no mutations in the proband, father or mother.

In one homozygous case, a six-year-old female patient presented with corneal opacities. Bilateral dense and confluent opacities with peripheral cornea non-involvement in the stromal layer were observed by slit-lamp examination (Figure 3A). Her father showed bilateral star- and disc-shaped opacities, which were less severe than those in his daughter (Figure 3B). Her mother had clear corneas. Sequencing of *TGFBI* revealed a homozygous R124H mutation (CGC→CAC), a heterozygous R124H mutation, and no mutations in the proband, father or mother (Figure 3C).

#### Lattice corneal dystrophy

Four families and five individuals were identified with lattice corneal dystrophy (LCD). The mean age of this group was 52 years (±15 years, range 22–80). Ten patients in this group had type 1 LCD, including two familial cases carrying the R124C mutation (all heterozygous form). There were two variant mutations, L527R at exon 12 in one family, and one novel mutation, P542R in exon 12 in one family (all heterozygous form). In the type 1 LCD, in most cases the onset of symptoms (most commonly decreased vision) started during the fourth decade of life. Recurrent erosions were also frequent. Thin branching refractile lines and/or subepithelial, whitish, ovoid dots usually appeared at a young age. The lines start centrally and superficially, spreading centrifugally and deeply. A diffuse stromal, ground-glass haze usually develops later, accompanied by recurrent erosions.

**Figure 4:**
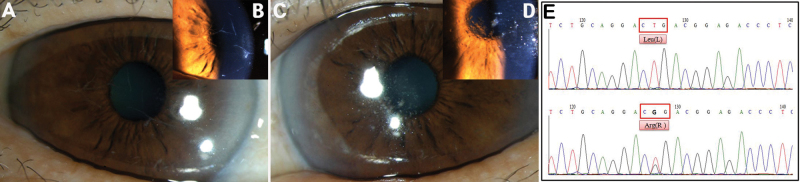
Phenotype of variant lattice corneal dystrophy (L527R mutation) with asymmetric corneal opacity in eye laterality, in a 67-year-old female. **A**, **B**: Several bifurcating, thick lattice lines in the superficial stroma of the right cornea. **C**, **D**: Discrete and nodular opacities were noted in the deep stroma of the central cornea. **E**: DNA sequence analysis of the *TGFBI* gene (upper) and a heterozygous point mutation, CTG→CGG (c.1580 T>G: Leu527Arg in exon 12) in codon 527 (bottom).

**Figure 5:**
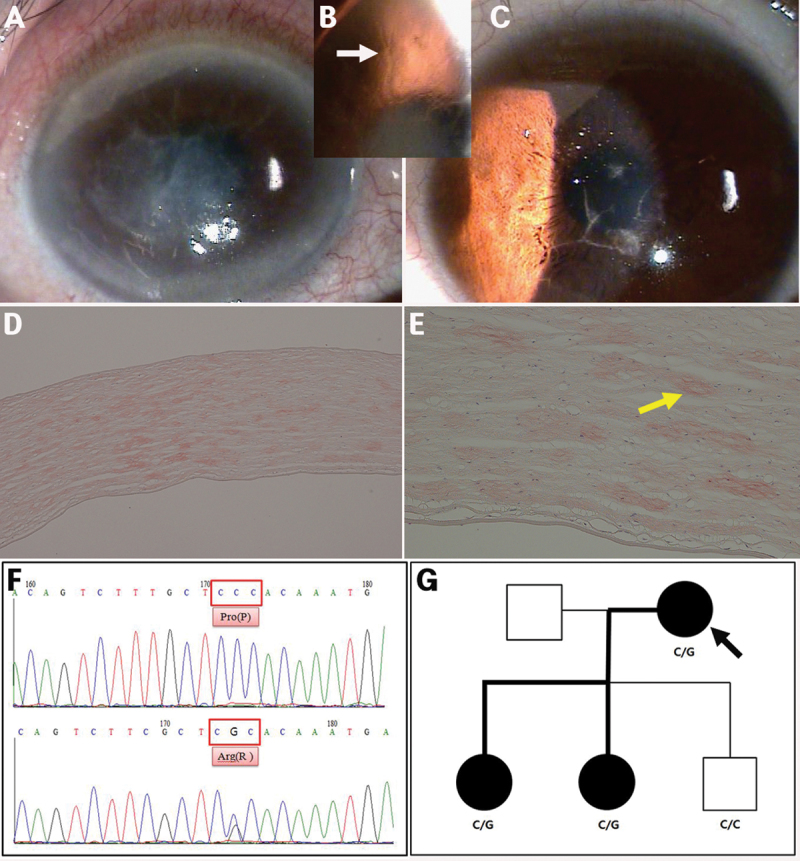
Family with variant lattice corneal dystrophy (novel mutation R542P). **A**, **B**: An 87-year-old female (proband), relatively thick lattice lines (white arrow) that extended from limbus to limbus in the superficial and deep stromal layers. **C**: A 55-year-old male (daughter of proband), lattice lines discrete in the superficial stroma of the central cornea. **D**, **E**: Histopathology (proband, 40× and 100×, respectively), Congo red-positive deposits in almost an entire corneal stroma layer. **F**: DNA sequence analysis of the *TGFBI* gene (upper) and a heterozygous point mutation, CCC→CGC (c.1625 C>G: Pro542Arg in exon 12) in codon 542 (bottom). **G**: Pedigree, heterozygous P542R mutation in the proband and her two daughters.

A 67-year-old Korean female patient was referred to our hospital due to gradual impairment of vision over the previous year. She had not experienced corneal trauma, nor did she have any family members with corneal problems. On her first visit, her corrected visual acuity was 20/50 OD and 20/32 OS. A slit-lamp examination revealed several bifurcating, thick lattice lines in the superficial stroma of the right cornea (Figure 4A,B). These lines were located on the mid-periphery and extended to the central stroma. In the left cornea, discrete and nodular opacities were noted in the deep stroma of the central cornea (Figure 4C,D). Lattice lines were not identified in the left eye and there were no nodular deposits in the right eye. The patient also had cataracts that had resulted in mild to moderate cortical opacity in both eyes. Heterozygous point mutations were detected, CTG→CGG (c.1580 T>G: Leu527Arg in exon 12), in codon 527 of *TGFBI* (Figure 5E). Slit-lamp examination of her daughter revealed no corneal lesions, and analysis of the daughter’s DNA revealed no specific mutation. After both of the patient’s crystalline lenses were extracted and intraocular lenses were inserted into both eyes, her visual acuity improved to 20/20 OD and 20/25 OS.

An 80-year-old Korean female patient was referred to our hospital due to ocular pain and gradual impairment of vision in the right eye over the previous 20 years. She had not experienced ocular trauma, but underwent corneal transplantation of the left eye 10 years ago due to the same symptoms in her right eye. Her corrected visual acuity was finger counting 30-cm OD and 20/200 OS. Relatively thick lattice lines extending from the limbus in the superficial stromal layers to the limbus in the deep stromal layers were observed in the right eye by slit lamp biomicroscopy. Epidefect and stromal haze were detected at the center of the right cornea (Figure 5A,B). The patient’s two daughters also showed corneal lattice lines, but these were less severe than the proband (Figure 5C,G). Examination of one corneal button of the proband confirmed the deep location of Congo red-positive deposits in the corneal stroma (Figure 5D,E). After informed consent, *TGFBI* analysis was performed using peripheral blood leukocytes. We detected a heterozygous point mutation, CCC→CGC (c.1625 C>G: Pro542Arg in exon 12) in codon 542 of *TGFBI* (Figure 5F).

## Discussion

In this first comprehensive report of *TGFBI* mutations in a Korean population, we detected three corneal dystrophy types (TBCD, GCD2, and LCD). When we consider the large number of subjects enrolled in this study, fewer dystrophy types related to *TGFBI* were observed compared to other studies. We did not find GCD1 and Reis-Bücklers corneal dystrophy (RBCD), which have been observed in other populations (Table 3). This may be due to the geographically isolated nature of Korea and its distinct population. GCD2 predominated over Lattice and Bowman’s layer dystrophies. We identified five distinct mutations responsible for *TGFBI* corneal dystrophies (p.R555Q, p.R124H, p.R124C, p.L527R, and p.P542R). The p.L527R mutation has been reported mainly in Japan [15], while P542R is novel mutation.

**Table 3:** Mutational analysis of *TGFBI* in defined populations (Korea, Mexico, New Zealand, Polish, Taiwan, and Ukraine).

**Population**	**Corneal dystrophies (*TGFBI* mutations)**
Korea [this study]	* GCD2 (R124H), TBCD (R555Q), LCD1 (R124C), Variant LCD (L527R, P542R)
Polish [9]	* LCD1 (R124C), GCD1 (R555W), GCD2 (R124H), TBCD (R555Q), Variant LCD (H626R)
Ukraine [10]	* LCD1 (R124C), GCD1 (R555W)
Mexico [11]	* Variant LCD (H626R), GCD1 (R555W), GCD2 (L550P), atypical GCD (L550P/H626R, V113I), PCA (A546D)
Taiwan [12]	* GCD2 (R124H), GCD1 (R555W), TBCD (R555Q), LCD1 (R124C), Variant LCD (A546D)
New Zealand [13]	* GCD1 (R555W), CDB (H626P), ** LCD

GCD1: Granular corneal dystrophy, type 1; GCD2: Granular corneal dystrophy, type 2; LCD1: Classic corneal dystrophy; Variant LCD: Variant lattice corneal dystrophy; TBCD: Thiel-Behnke corneal dystrophy; CDB: Corneal dystrophy of Bowman layer); *: Most common, **: No pathogenic *TGFBI* sequence variants were detected.

Patients with corneal opacities at the level of Bowman’s layer have been previously diagnosed as having corneal dystrophy of the Bowman layer (CDB), but some were thought to have a superficial variant of granular corneal dystrophy [16]. RBCD and TBCD are typically characterized by geographic opacities and honeycomb-shaped opacities, respectively. Most patients reported to have RBCD had the R124L mutation and those reported to have TBCD had the R555Q mutation [17,18]. Our data indicated that all patients with Bowman’s layer dystrophy carried the R555Q mutation (heterozygous form), but not R124L. These patients would have the characteristic presentation of honeycomb corneal opacities at the Bowman’s layer and superficial stroma, which were correlated with the R555Q mutation at a young age. However, the opacities united, forming denser and larger opacities, and progressed to deep stromal layers and the periphery of the cornea with advancing age, making it impossible to distinguish from Reis-Bucklers or other types of corneal dystrophy. Onset of symptoms or signs were evident in childhood; indeed, an 11-month-old female patient showed bilateral, irregularly shaped, scattered opacities of the cornea with the R555Q mutation.

Granular corneal dystrophy type 2 (GCD) (Avellino corneal dystrophy; OMIM 607541) is inherited as an autosomal dominant form with very high penetrance [19]. This disease has clinical and histologic features of both granular and lattice dystrophy. Hyaline granular deposits are the earliest and most common manifestations [4,20]. Amyloid lattice lesions were present in the deeper stroma in some patients with granular lesions. Older patients developed an anterior stromal haze between deposits, which impaired visual acuity. In our study, the onset of symptoms (most commonly decreased vision) occurred in the fifth or sixth decades, and initial signs (incidentally discovered corneal opacities) were evident in the third or fourth decades. In the early stages, the signs were subtle: tiny whitish dots. As the condition progressed, star-shaped stromal opacities or lattice lines appeared. Granular opacities formed via gradual aggregation of the surrounding fine deposits.

Various refractive surgeries, including photorefractive keratectomy (PRK), laser in situ keratomileusis (LASIK), and laser epithelial keratomileusis (LASEK), have become popular techniques for correction of refractive errors worldwide. However, several cases that were exacerbated after refractive surgery in patients with GCD2 have been reported [21,22]. The morphological features of corneal deposits in patients with a history of laser refractive surgery differed from those in other patients. Deposits after laser ablation appeared mainly within the ablation zone and exhibited diffuse, confluent, white, and small opacities. In most cases, these opacities coexisted with discrete and granular opacities that may not be associated with the refractive surgery.

In one homozygous case, a six-year-old female patient presented with dense and confluent opacities with non-involvement of the peripheral cornea in the stromal layer. Her father showed bilateral star- and disc-shaped opacities, but these less severe than those in his daughter. Interestingly, sequencing of the *TGFBI* gene revealed a homozygous R124H mutation (CGC→CAC), a heterozygous R124H mutation, and no mutations in the proband, father, or mother. Therefore, this may be an area of high vulnerability of mutation in the Korean population. Spontaneous mutations in *TGFBI* have been reported previously, including an R124L mutation in two patients with RBCD and an R555Q mutation in two families with CDB [23,24]. In our case, a spontaneous R124H mutation was added to an already mutated allele, changing a heterozygous to a homozygous form.

LCD1 is characterized by a network of delicate interdigitating filaments within the corneal stroma. The disease usually begins in the first decade of life with symptoms of recurrent painful epithelial erosions. Lattice lines and diffuse opacification of the central cornea develop gradually after the erosions and amyloid accumulations. The most common mutation in the TGFBI gene in patients with LCD1 is R124C. Numerous forms of atypical LCD have been reported to be caused by mutations at P501T, V505D, L518P, I522N, L527R, V539D, A546D, A546T, P551Q, L569R, H572R, V625D, or H626R [25-30]. In type 1 (classic) LCD, the onset of symptoms (most commonly decreased vision) most often occurred within the fourth decade. Recurrent erosions are also frequent. Thin branching refractile lines and/or subepithelial, whitish, ovoid dots usually appeared at a young age. The lines start centrally and more superficially, spreading centrifugally and deeply. A diffuse stromal, ground-glass haze usually develops later, accompanied by recurrent erosions. In this study, there were two interesting cases. First, a 67-year-old Korean female presented with several bifurcating, thick lattice lines in the superficial stroma of the right cornea. In the left cornea, discrete and nodular opacities were noted in the deep stroma of the central cornea. We found no lattice lines in the left eye or nodular deposits in the right eye. We detected a heterozygous point mutation, CTG→CGG (c.1580 T>G: Leu527Arg in exon 12), in codon 527 of *TGFBI* (Figure 5E). Recent studies have revealed that late-onset lattice corneal dystrophy is caused by several mutations in *TGFBI*. Among these, L527R has been reported mainly in Japan and has various clinical manifestations, including late onset, sporadic occurrence, and asymmetric corneal opacities [27]. It is not clear why a single mutation can cause several corneal opacity patterns. However, atypical corneal opacity patterns may be characteristic of late-onset lattice corneal dystrophies. Other than the phenotype in this case, this type of mutation seems to be caused principally by accidental replication errors during cell division that may escape both the proofreading function of DNA polymerase and the DNA mismatch-repair process, with an estimated frequency of 1 in 10^9^ to 10^10^ base pairs per cell division [31]. Hence, these mutations are predisposed to be much less frequent and tend to become a founder mutation. It has been reported that the p.L527R mutation is descended from a founder mutation that occurred in a single Japanese ancestor [32]. Given that we identified a Korean patient with lattice corneal dystrophy due to the L527R mutation in *TGFBI*, although this mutation is very rare, it may occur in individuals of nationalities other than Japanese. Second, a novel *TGFBI* mutation, P542R was identified in our case. It caused relatively thick lattice lines that extended from the limbus of the superficial stromal layers to the limbus in the deep stromal layers. Examination of one corneal button of the proband confirmed the depth of Congo red-positive deposits in the corneal stroma. A heterozygous point mutation, CCC→CGG (c.1625 C>G: Pro542Arg in exon 12), was detected in the proband and two daughters.

In conclusion, we report here phenotype-genotype correlations and novel mutations in a Korean population with *TGFBI*-related corneal dystrophies. A relatively large group of families and patients were analyzed compared to other studies, and the data suggested that the phenotype changes over time. R124H in GCD2 was the most common mutation; however, GCD1 and Reis-Bucklers corneal dystrophy were not found. There were many laser refractive surgery-induced corneal opacities in GCD2 patients, and in one case a spontaneous R124H mutation was added to an already mutated allele, resulting in a change from a heterozygous to a homozygous form. A novel mutation, P527T, was identified in LCD.
